# Studying of the promotion mechanism of *Bacillus subtilis* QM3 on wheat seed germination based on β-amylase

**DOI:** 10.1515/biol-2020-0062

**Published:** 2020-10-14

**Authors:** Ya-Jing Li, Qing-Ping Hu

**Affiliations:** School of Life Sciences, Shanxi Normal University, 1 Gongyuan Street, Linfen City, 041004, China

**Keywords:** *Bacillus subtilis* QM3, early seed germination, β-amylase

## Abstract

The response of β-amylase in early stage germination of wheat seeds to *Bacillus subtilis* QM3 was mainly focused on to elucidate the promotion mechanism of *B. subtilis* QM3. The results showed that the changes in apparent activity of amylase and endosperm liquefaction after the strain QM3 treatment were much more obvious than that of the control group; the activity of β-amylase treated with the different concentrations of the strain QM3 increased significantly (*P* < 0.05) by 4% (10^7^ CFU/mL) and 18.5% (10^6^ CFU/mL) at the germination 6 h. Moreover, after presoaking with α-cyclodextrin, the activity of β-amylase increased significantly (*P* < 0.05) by 18.5% (10^7^ CFU/mL) and 23.4% (10^6^ CFU/mL) at the same stage of germination; the electrophoretogram of β-amylase isoenzymes showed that there is a growing trend in brightness and width of the band during the early germination from 3 to 6 h of wheat seed treated by the strain QM3 (10^6^ CFU/mL). The increase in activity and isoenzyme expression of β-amylase may be one of the important reasons to promote the germination of wheat seeds after being treated by *B. subtilis* QM3.

## 1 Introduction

Seed germination begins the crop life cycle, which is closely related to seed sprouting and subsequent plant growth and development, and ultimately affects the grain yield and quality. Timely germination and uniform emergence are pivotal determinants in modern agricultural production systems for many crops [1]. As an important part of life cycle, sprouting is the basis of crop growth. Wheat (*Triticum aestivum* L.) is one of the three most widely cultivated cereal crops in the world, and there is an intensive need to increase wheat production to meet the population demands [1,2]. During germination of wheat seeds, various hydrolysis enzymes are synthesized and secrete mobilization reserves. Starch is one of the principal storage reserves in the wheat seed. The activity and content of amylase are the key factors of wheat seed germination, even though the germination of grain seeds is affected by many factors [3].

β-amylase (a-1,4-glucan maltohydrolase, E.C. 3.2.1.2) has been closely observed during the wheat seed germination since the 1980s. Its major physiological function is to work as an exoamylase by catalyzing the liberation of β-anomeric maltose from the nonreducing ends of starch [4]. The extent of β-amylase activity probably determines the germination ability of cereal seeds. Amylase occurs as many isozymes in plants. Studies have shown that varieties with a faster germination rate have more isozyme bands than those with a slower germination rate, indicating that isozymes have a certain relationship with the seed germination rate [5].

*Bacillus subtilis* QM3 is a plant growth-promoting rhizobacterium isolated from the Qinghai yak [6,7]. It has a high reproductive rate, and a previous study showed that *B. subtilis* QM3 had an auxo-action on wheat seed germination and has an obvious effect on the growth of tomato [7]. Further research is necessary to clarify the response of β-amylase in the wheat seed germination after treatment by *B. subtilis* QM3.

The purpose of this study was to investigate the effects of *B. subtilis* QM3 on the early germination of wheat seeds under pretreatment with a β-amylase-specific inhibitor, α-cyclodextrin, on the amylase, epigenetic activity of amylase, endosperm liquefaction, amylase activity, and amylase isoenzyme starch in the early stage of wheat seed germination, and to promote plant germination from physiological and biochemical aspects. Protein aspects provide some theoretical guidance, which lays a foundation for *B. subtilis* QM3 to promote crop germination and further use in agricultural production practices.

## 2 Materials and methods

### 2.1 Media

The commonly used medium in this investigation was the beef extract-peptone containing peptone 1.0%, beef extract 0.3%, sodium chloride 0.5%, and agar 1.5–2.0%, pH 7.4–7.6, sterilized at 121°C for 20 min.

### 2.2 Bacterial suspension preparation

*B. subtilis* QM3 used in the present study came from the Microbiological Laboratory, School of Life Sciences, Shanxi Normal University [7]. A culture of *B. subtilis* QM3 was obtained by transferring colony of the activated culture plate into a 250 mL flask containing 100 mL beef extract–peptone medium and shaking in an orbital shaker at 200 rpm at 37°C for 3 days. It was then diluted in sterile water in order to reach an OD_600 nm_ of 0.8 (10^8^ CFU/mL *B. subtilis* QM3). The liquid bacterial suspension was diluted 10 times and 100 times to reserve [8].

### 2.3 Seed materials

Wheat seeds (Linhan No. 9) were used from the Shanxi Academy of Agricultural Sciences. Healthy seeds of similar size and mass were selected. Before sprouting, the seeds were surface-sterilized in 5% sodium hypochlorite for 10 min and rinsed in distilled water [9].

Standard germination was conducted according to Ma et al. [10], using 50 seeds per replicate and three replicates. Seeds were incubated in growth cabinets at 25°C in the dark for 12 h and 25°C in the light for 12 h. The germinated seeds were counted daily and the germination percentage was recorded. Germination is based on the seed breaking through the seed coat.

\text{Germination}\hspace{.5em}\text{percentage}\hspace{.5em}(\text{GP})=(Gt/T)\times 100 \%

where *Gt* is the number of germinations in *t* days and *T* is the total number of seeds used in the test.

### 2.4 Apparent enzyme activity and the liquefaction of endosperm

Seeds were divided into two large groups that soaked in sterile water (X) or α-cyclodextrin solution (Y) for 3 h, and then transferred to Petri dishes for germination. Each large group was placed in four Petri dishes as four small groups, two large groups, respectively, cultivated with sterile water (CK) (1), 10^7^ CFU/mL *B. subtilis* QM3 (2), 10^6^ CFU/mL *B. subtilis* QM3 (3), and sodium nitroprusside (SNP) (4) for 12 h. After that, the apparent enzyme activity and the liquefaction of endosperm were evaluated, and β-amylase activity and β-amylase isoenzyme samples were taken every 3 h. The seedlings grew in a constant temperature incubator (25°C, 12 h, and 55% relative humidity) [8]. The experiment was repeated three times.

Following Ikeda’s method [11], wheat seeds of different treatments were cut horizontally, and half of the seeds containing germs were inoculated on the medium (10 mmol/L acetate acid buffer pH 5.3, containing 2% agar, 0.2% boiled soluble starch, and 2 mmol/L CaCI_2_). After incubation at 25°C for 12 h, it was stained with 5-fold diluted mother liquid iodine. The bright zone around the seed on the medium represented the size of the apparent amylase activity. The larger and brighter the halo, the stronger the apparent amylase activity.

Another half of the seeds without embryos were inoculated on medium (10 mmol/L acetic buffer pH 5.3, containing 2% agar and 2 mmol/L CaCl_2_) at 25°C for 24 h and the liquefactions or dissolutions of endosperm were observed continuously.

### 2.5 Assay of β-amylase activity

#### 2.5.1 Extraction of the enzyme

One gram of wheat seeds was put in a mortar, a small amount of quartz sand and 2 mL of distilled water were added and then ground into a homogenate. The homogenate was transferred to a centrifugal tube with all residues that were washed with distilled water. The extract was placed at room temperature for 15–20 min and stirred every few minutes to make it fully extracted [12]. The homogenate was centrifuged once at 3,000*g* for 10 min to collect the supernatant. Then the supernatant was poured into a 100 mL volumetric flask and distilled water was added to a constant volume to the scale. Then by absorbing 10 mL of the alpha amylase solution and putting it into a 50 mL volume bottle, the amylase dilution solution was determined by distilled water to the scale.

#### 2.5.2 Determination of enzyme activity

Total β-amylase activities in seeds germinated into different concentrations of bacterial fluid were measured daily by the method of 3,5-dinitrosalicylic acid (DNS). *A*_540 nm_ was measured by a spectrophotometer of Shanghai Jinghua Science & Technology Instruments Co., Ltd. All measurements were repeated in triplicate. The amount of maltose liberated was extrapolated from the maltose standard curve. One unit (U) of amylase activity was defined as the amount of enzyme necessary to produce 1 mg of maltose per minute under the optimum conditions [13].

### 2.6 Assay of β-amylase isozyme

#### 2.6.1 Preparation of amylase extract

Half a gram of wheat seeds was ground with 5 mL of phosphate buffer (pH 7.5) in an ice bath. The homogenate was centrifuged at 10,000*g* for 30 min. The supernatant was collected in an Eppendorf tube, and that was the total amylase solution. Then HCl 4 mol/mL was added to the total amylase to make its pH less than 4 and then the solution was centrifuged at 10,000*g* for 30 min. The supernatant obtained was designated as β-amylase.

#### 2.6.2 Isozyme extract by native polyacrylamide gel electrophoresis (PAGE)

Amylase electrophoresis was assayed by the photochemical method described by Wu et al. with a slight change [4]. Ten micrograms of protein (10 μL) in each extract from each variety were mixed with a 5:1 volume of sample buffer (150 mmol/mL Tris–HCl pH 6.8, 20% glycerol, and 0.001% bromophenol blue) without boiling, and then loaded onto a native-PAGE gel which had a 10% separation gel with acrylamide:bisacrylamide at a ratio of 29:1 in 150 mmol/mL Tris–HCl pH 6.8 and a 5% stacking gel using 150 mmol/mL Tris–HCl pH 8.8. All gel components contained no SDS. Electrophoresis was carried out at 80 V per gel for 1 h for the stacking step and 110 V per gel for 2.5 h for the resolving step in running buffer (25 mmol/mL Tris and 400 mmol/mL glycine), until the dye had run to the edge of the gel.

### 2.7 Statistical analysis

Data were analyzed statistically by variance using the SPSS 11.0 statistical software packages. Differences were assessed by the Duncan’s ANOVA test, and they were significant with *P* < 0.05 [14].

## 3 Results

### 3.1 Germination rate

**Figure 1:**
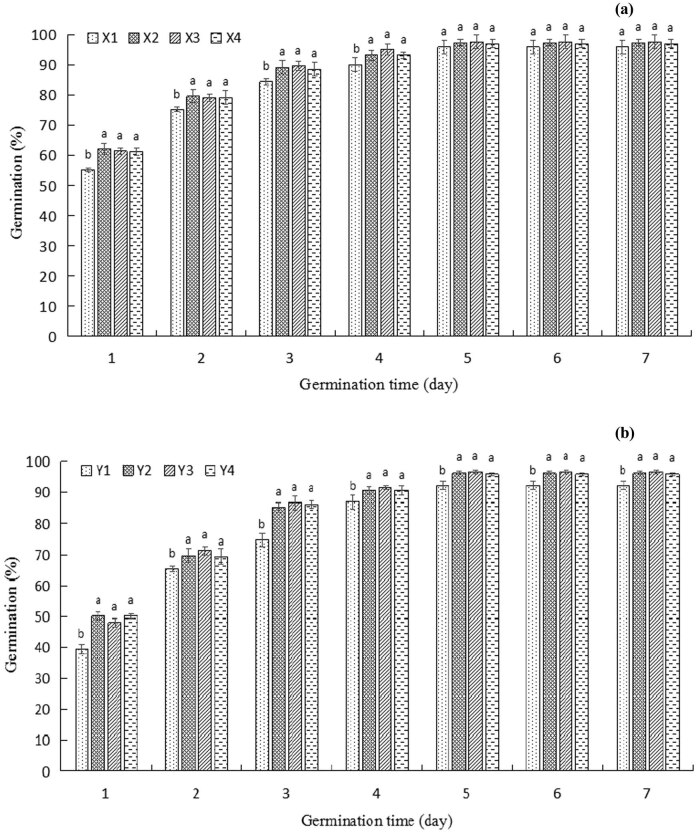
Effect of *B. subtilis* QM3 and α-cyclodextrin on the germination rate of wheat seeds. (a) The first group treated with sterile water under different treatments including X1, X2, X3, and X4, which represent the control group (sterile water, X1), 10^7^ CFU/mL *B. subtilis* QM3 (X2), 10^6^ CFU/mL *B. subtilis* QM3 (X3), and SNP (X4), respectively. (b) The second group treated with α-cyclodextrin under different treatments including Y1, Y2, Y3, and Y4, which represent the control group (sterile water, Y1), 10^7^ CFU/mL *B. subtilis* QM3 (Y2), 10^6^ CFU/mL *B. subtilis* QM3 (Y3), and SNP (Y4), respectively. Different lowercase letters represent a significant difference between different treatments (*P* < 0.05).

On the whole, the germination rate of the first 5 days showed an increasing trend, and the germination rate of each treatment group did not change significantly by the 6th day. Compared to the control group X1, X2, X3, and X4, all have significant effects on promoting wheat germination (*P* < 0.05); especially in the first 1–3 days of germination, the X2 group experienced the largest increase of germination rate to 12.68% (Figure 1a). These results suggested that the strain QM3 could improve the germination rate of wheat seeds in early stage germination.

α-Cyclodextrin is a specific inhibitor of β-amylase. Figure 1a and b show that the germination rate of wheat seeds soaked in α-cyclodextrin (Y1) was reduced by 10–15%, compared to X1 during the first three days. However, compared with Y1, the germination rates of Y2, Y3, and Y4 were significantly higher on the third day, the treatment Y3 was higher by 16.06% (Figure 1b). In short, α-cyclodextrin showed obvious inhibition on the germination rate of wheat seeds, while the strain QM3 could effectively alleviate the inhibition of cyclodextrin on the germination rate of wheat seeds in early stage germination.

### 3.2 Changes in apparent amylase activity

**Figure 2:**
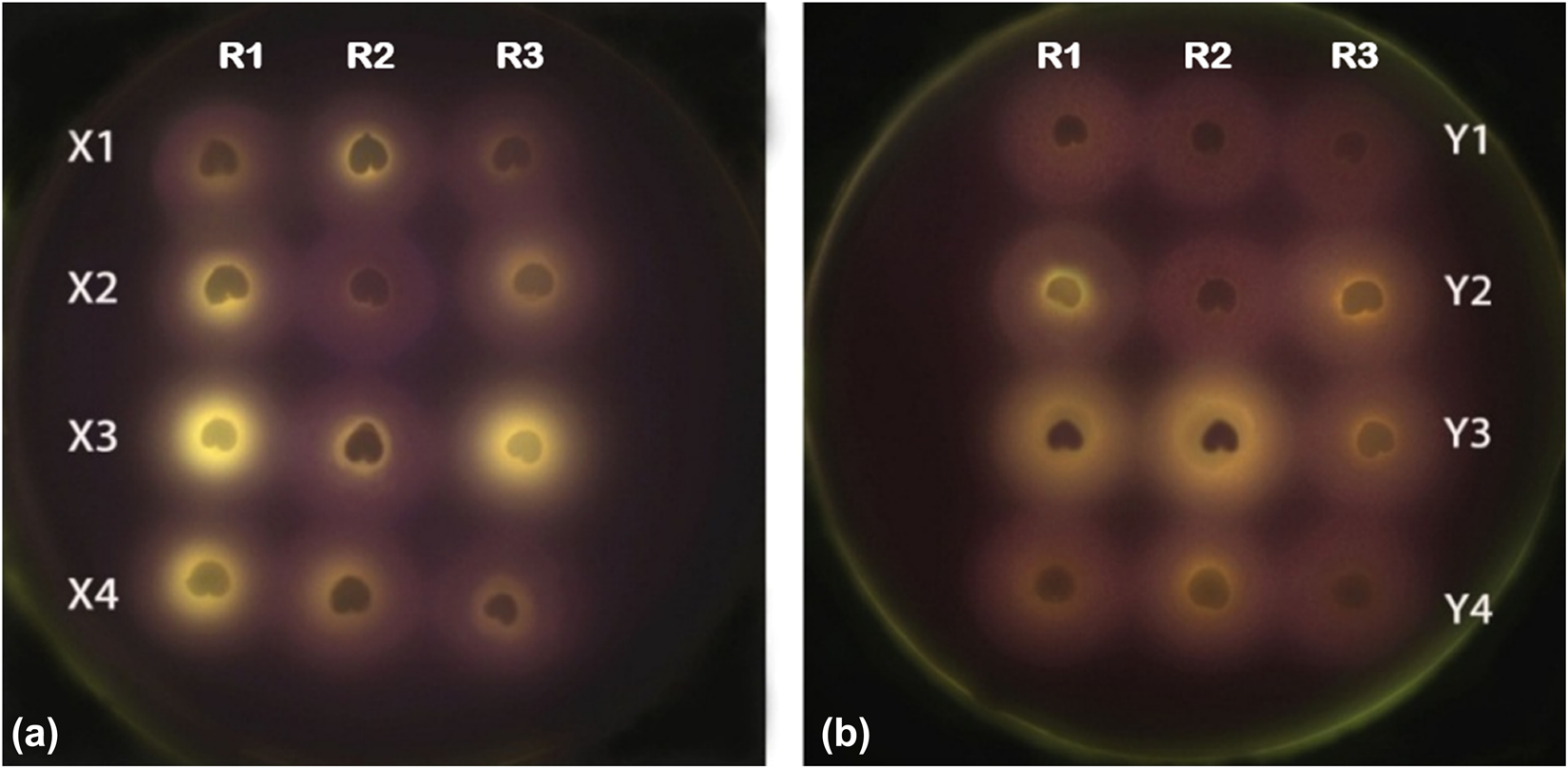
The apparent amylase activity of half seeds with embryo. (a) The first group treated with sterile water under different treatments including X1, X2, X3, and X4, which represent the control group (sterile water, X1), 10^7^ CFU/mL *B. subtilis* QM3 (X2), 10^6^ CFU/mL *B. subtilis* QM3 (X3), and SNP (X4), respectively. (b) The second group treated with α-cyclodextrin under different treatments including Y1, Y2, Y3, and Y4, which represent the control group (sterile water, Y1), 10^7^ CFU/mL *B. subtilis* QM3 (Y2), 10^6^ CFU/mL *B. subtilis* QM3 (Y3), and SNP (Y4), respectively. R1, R2, and R3 represent the same process repeated three times.

This experiment of the apparent enzyme activity is a qualitative method of determining the size of amylase activity. Each process was repeated three times, based on the overall result. It can be seen from the X group (Figure 2a) that for wheat seeds cultured with sterile water, the range of bright spots on the seeds on the agar plate was very small. On the contrary, for seeds cultured with X2 and X4, the size of bright spots on the seeds increased slightly, while for seeds cultured with X3, the size of bright spots on the seeds increased significantly compared with that of X1.

The same thing happens to the Y group (Figure 2b) soaked in α-cyclodextrin for 3 h, which can inhibit the activity of β-amylase. For seeds cultured with Y2 and Y4, the size of bright spots on the seeds increased slightly, while for seeds cultured with Y3, the size of bright spots on the seeds increased significantly compared with that of Y1. It shows that *B. subtilis* QM3 can obviously enhance the amylase activity of α-cyclodextrin inhibitor wheat seeds during early germination.

Exogenous NO has a strong stimulating effect on wheat seed germination by inducing a rapid increase in β-amylase activity and greatly promotes germination processes by enhancing the activities of amylase. Addition of SNP, an NO donor, was able to induce a rapid increase in β-amylase activity and stimulated seed germination under severe salt stress in wheat [13]. In agreement with this, we also observed that the halo of treatment 4 is brighter and wider than treatment 1’s for both group X and group Y. However, treatment 3 is more effective than treatment 4 in both large groups, which indicates that exogenous *B. subtilis* QM3 significantly enhanced the amylase activity in early stage germination of wheat seeds, especially when the concentration of the strain QM3 was suitable.

### 3.3 Results of liquefaction of endosperm

**Figure 3:**
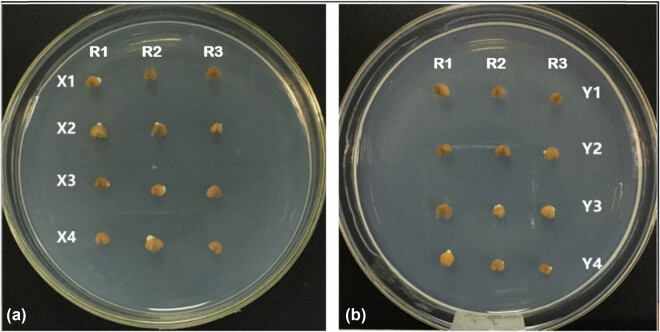
The liquefaction of endosperm of another half seeds without embryo. (a) The first group treated with sterile water under different treatments including X1, X2, X3, and X4, which represent the control group (sterile water, X1), 10^7^ CFU/mL *B. subtilis* QM3 (X2), 10^6^ CFU/mL *B. subtilis* QM3 (X3), and SNP (X4), respectively. (b) The second group treated with α-cyclodextrin under different treatments including Y1, Y2, Y3, and Y4, which represent the control group (sterile water, Y1), 10^7^ CFU/mL *B. subtilis* QM3 (Y2), 10^6^ CFU/mL *B. subtilis* QM3 (Y3), and SNP (Y4), respectively. R1, R2, and R3 represent the same process repeated three times.

In order to further study the mobilization mechanism of stored material in wheat seeds, we designed the chemical dissolution experiment of starch endosperm. It was different from the size of starch secretory plaque and the main storage material in the medium in that the treated seeds without embryos showed large-scale liquefaction or secretory dissolution of endosperm after inoculation, such as X2, X3, X4, Y3, and Y4. Among them, the treatment group showed the most obvious effect, while the control group showed only a small amount of liquefaction (Figure 3). The exogenous *B. subtilis* QM3 can also significantly increase the liquefaction endosperm in early germination of wheat seeds.

### 3.4 Determination of β-amylase activity

**Figure 4:**
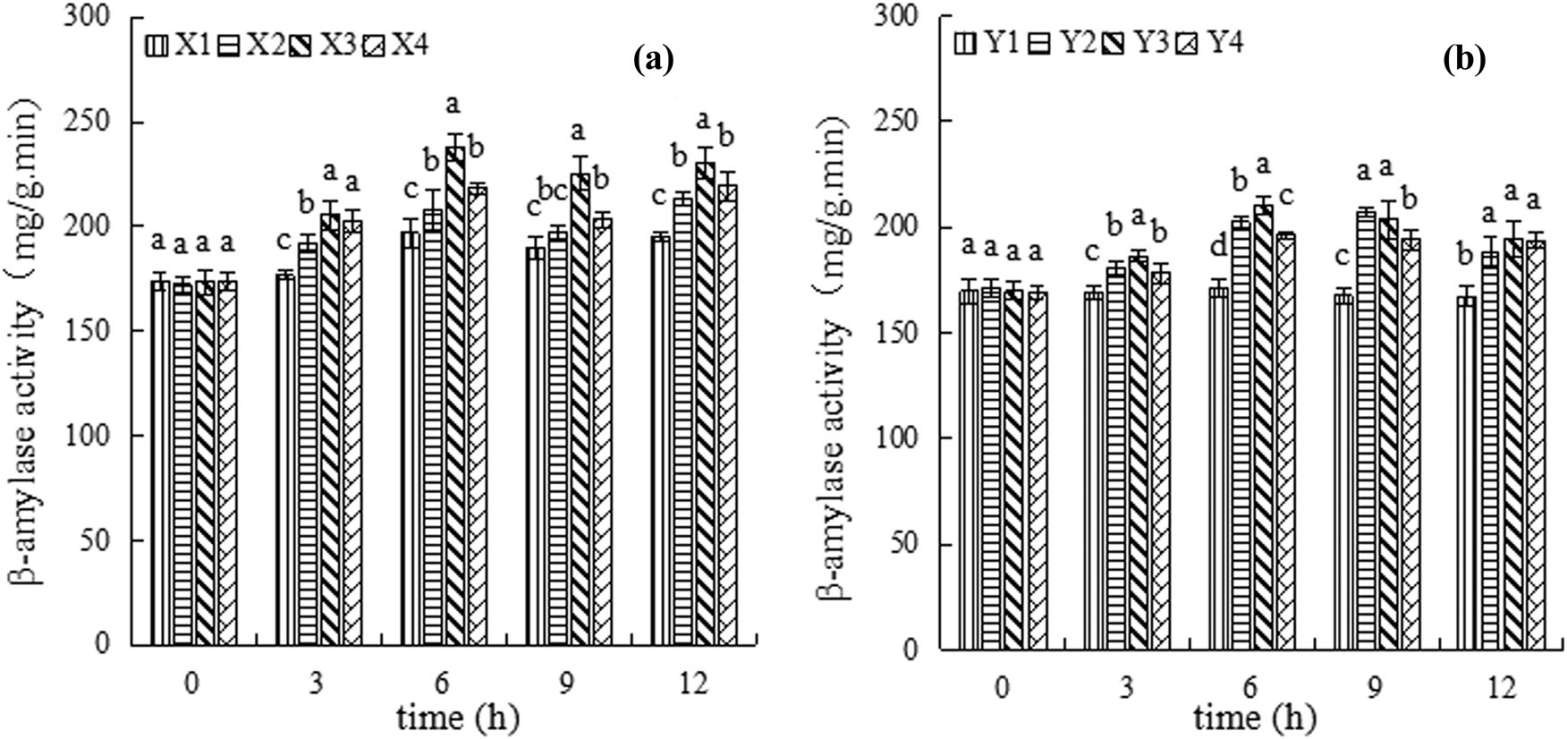
Changes in β-amylase activity during early germination of wheat seeds. (a) The first group treated with sterile water under different treatments including X1, X2, X3, and X4, which represent the control group (sterile water, X1), 10^7^ CFU/mL *B. subtilis* QM3 (X2), 10^6^ CFU/mL *B. subtilis* QM3 (X3), and SNP (X4), respectively. (b) The second group treated with α-cyclodextrin under different treatments including Y1, Y2, Y3, and Y4, which represent the control group (sterile water, Y1), 10^7^ CFU/mL *B. subtilis* QM3 (Y2), 10^6^ CFU/mL *B. subtilis* QM3 (Y3), and SNP (Y4), respectively. Different lowercase letters represent a significant difference between the treatment 1, 2, 3, and 4 under the same time (*P* < 0.05).

There are still two large groups of group X (soaked in sterile water) and Y (soaked in β-cyclodextrin). All the β-amylase activity samples were determined from germination 0 to 12 h. With the increase in germination time, the β-amylase activity of wheat seeds first increased and then decreased. After the treatment of wheat seeds with different concentrations of *B. subtilis* QM3, the β-amylase activity of wheat seeds increased in different extents. The β-amylase activity samples of X2, X3, and X4 were significantly increased, compared with that of X1 without exception. The activity at germination 3 h showed a significant increase in X2, X3, and X4, and there was a small increase at 9 and 12 h in these three treatments. When the germination wastes 6 h that is relatively large and the increase in rates reached the largest value. Compared with X1, the β-amylase activity samples of X2, X3, and X4 increased significantly by 4%, 18.5%, and 7.1%, respectively (*P* < 0.05) (Figure 4a). The results showed that the exogenous *B. subtilis* QM3 (X2 and X3) could increase the activity of β-amylase during wheat seed early germination, which was beneficial to seed germination like SNP. Especially, the treatment X3 was the most effective.

In group Y it can be observed that with time, the β-amylase activity first increased and then became stable when the samples were pretreated by β-cyclodextrin, which is an inhibitor of the β-amylase. Obviously, β-amylase activities of Y2, Y3, and Y4 were higher than that of Y1. But the increase in trend was milder than that of group X and its maximum was reached at the same germination time as X. Compared with Y1, β-amylase activities of Y2, Y3, and Y4 increased significantly by 18.5%, 23.4%, and 14.9%, respectively (*P* < 0.05) at the germination 6 h (Figure 4b).

Combining the results of group X and group Y, it can be deduced that *B. subtilis* QM3 could improve the β-amylase activity effectively during early germination of wheat seeds and promote the process of germination. As a factor promoting germination, the efficacy of bacteria is even greater than that of SNP.

### 3.5 Analysis of β-amylase isoenzymes

**Figure 5:**
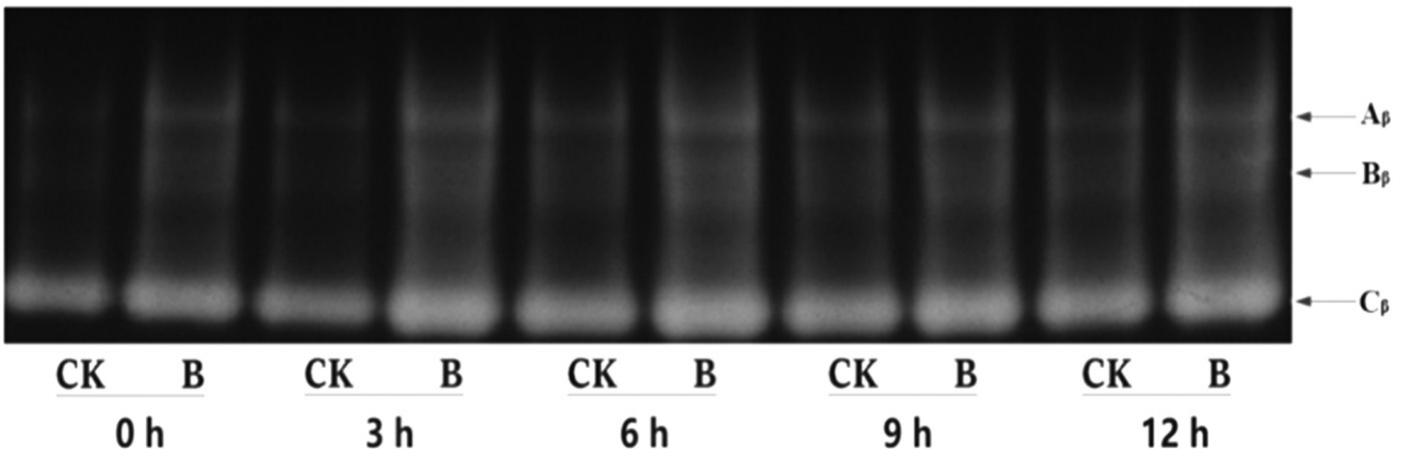
Effect of *B. subtilis* QM3 on β-amylase isoenzymes in early stage germination of wheat seeds. CK and B represent sterile water treatment and *B. subtilis* QM3 (10^6^ CFU/mL) treatment, respectively. A_β_, B_β_, and C_β_ are three different β-amylase isozyme bands. 0, 3, 6, 9, and 12 h are germination times of seeds.

Changes in the number and brightness of electrophoretic bands of β-amylase isoenzymes during wheat seed germination can largely reflect the β-amylase activity and content. According to the results of the above experiments, we selected the *B. subtilis* QM3 (10^6^ CFU/mL) treatment (B) and the sterile water treatment (CK) for electrophoresis to analyze the β-amylase isoenzyme (Figure 5).

There were three isozyme types, A_β_, B_β_, and C_β_, which were observed in the gels, and most of the β-amylase activity could be attributed to C_β_. The band A_β_ was a narrow band, and the width of the B_β_ and C_β_ bands was relatively larger. At different germination times, the number and brightness of the bands of the B group were different from those of the CK group. With the prolongation of germination time, the width and brightness of the bands increased. The number and brightness of the bands of the B group at germination 6 h were significantly different from those of the other germination stage. This explains that treatment B can increase the activity and content of β-amylase isoenzyme during the early germination of wheat seeds. However, at germination 6 h, it had the most obvious promotion, attributed especially to the specific B_β_ and C_β_ bands of β-amylase isoenzyme*. B. subtilis* QM3 may increase the expression of β-amylase isoenzyme by increasing the brightness and width of the B_β_ and C_β_ bands, thereby promoting wheat seed germination (Figure 5).

## 4 Discussion

It is well known that a wheat seed germination greatly depends on its β-amylase activities [14] and increasing β-amylase activities by exogenous 10^6^ CFU/mL of *B. subtilis* QM3 was important to the efficient activity. β-Amylase is a key enzyme in the process of seed sprouting [15] and can reflect the seed germination vigor. Moreover, the activity of β-amylase affects the germination rate and seedling survival rates of wheat, and the activity of β-amylase and the speed of plant seed germination have a certain relationship [16]. In this work, we have provided evidence for an involvement of *B. subtilis* QM3 (10^6^, 10^7^ CFU/mL) in the rapid induction of β-amylase in early stage germination of wheat seeds.

The germination stimulation by exogenous modulators is greatly attributed to increasing amylase expression and activity [14]. In the present study, similar results were obtained. *B. subtilis* QM3 as an exogenous modulator can significantly increase the activity of β-amylase (Figure 4) and the expression of β-amylase isoenzyme (Figure 5) during the early stage germination. This indicated that *B. subtilis* QM3 can promote the germination of wheat seeds by increasing the activity of amylase.

Amylase isoenzyme can be used as a biochemical indicator at different stages of seed development. There are many amylase isoenzymes in plants, and the band of amylase isoenzyme has a certain relationship to the rate of seed germination, which can affect the amylase activity [4]. The number and brightness of bands represent the content of the enzyme. The results of this experiment showed that with the increase in wheat seed sprouting time, the brightness of the bands of the β-amylase isoenzyme increased. After soaking seeds with *B. subtilis* QM3 (10^6^ CFU/mL), β-amylase isoenzyme bands increased and broadened, which enhanced the expression activity of the enzyme bands.

In summary, all of the β-amylase activities were significantly increased by exogenous *B. subtilis* QM3 (10^6^ CFU/mL) and there are significant differences between the strain and SNP. It must be mentioned, however, that germination involves the mobilization of reserves as a whole, rather than being an event associated with one or two enzymes [5]. It would be interesting to study the further regulatory mechanisms of early germination controlled by *B. subtilis* QM3. Moreover, the results suggested that β-amylase activity could be used as an index for cereal germination potential, due to the fact that it was associated with initiation of germination of seedling growth at a later stage of germination. Therefore, the modulation of *B. subtilis* QM3 on proline might be involved in NO pathway, and the mechanism needs further investigation. Choosing the appropriate concentration of bacteria solution is more conducive to promoting the germination of wheat seeds increasing the crop yields. This phenomenon may be related to the combination of bacteria and wheat seeds, but the deeper reasons remain to be further studied.
